# Magma plumbing system and seismicity of an active mid-ocean ridge volcano

**DOI:** 10.1038/srep42949

**Published:** 2017-02-20

**Authors:** Florian Schmid, Vera Schlindwein, Ivan Koulakov, Aline Plötz, John-Robert Scholz

**Affiliations:** 1 Alfred-Wegener-Institute, Helmholtz Centre for Polar and Marine Research, Bremerhaven, Germany; 2 Trofimuk Institute of Petroleum Geology and Geophysics SB RAS, Novosibirsk, Russia Novosibirsk State University, Pirogova 2, 630090, Novosibirsk, Russia; 3 Institue of Geophysics and Geology, University of Leipzig, Germany

## Abstract

At mid-ocean ridges volcanism generally decreases with spreading rate but surprisingly massive volcanic centres occur at the slowest spreading ridges. These volcanoes can host unexpectedly strong earthquakes and vigorous, explosive submarine eruptions. Our understanding of the geodynamic processes forming these volcanic centres is still incomplete due to a lack of geophysical data and the difficulty to capture their rare phases of magmatic activity. We present a local earthquake tomographic image of the magma plumbing system beneath the Segment 8 volcano at the ultraslow-spreading Southwest Indian Ridge. The tomography shows a confined domain of partial melt under the volcano. We infer that from there melt is horizontally transported to a neighbouring ridge segment at 35 km distance where microearthquake swarms and intrusion tremor occur that suggest ongoing magmatic activity. Teleseismic earthquakes around the Segment 8 volcano, prior to our study, indicate that the current magmatic spreading episode may already have lasted over a decade and hence its temporal extent greatly exceeds the frequent short-lived spreading episodes at faster opening mid-ocean ridges.

Oceanic lithosphere is created at mid-oceanic ridges by a complex interplay of magmatic and tectonic processes. As spreading rates decrease the style and quantity of magmatism alter from nearly continuous magma extrusion along the axis of fast spreading ridges^1^, to discrete, widely spaced magmatic centres at the slowest spreading ridges^2^,^3^,^4^,^5^,^6^. Although magmatic centres are a common feature of slow spreading ridges (20–55 mm yr^−1^ full rate) and ultraslow-spreading ridges (<20 mm yr^−1^ full rate), there are fundamental differences between their representatives at both ridge classes. Studies of crustal thickness show that melt flux per segment length is constant at slow spreading magmatic centres^7^ but to explain the greatly thickened crust at ultraslow magmatic segments a melt flux exceeding the regional average is necessary^4^,^8^,^9^. Other unique features of magmatic centres at ultraslow ridges are the occurrence of unexpectedly strong and long lasting swarms of earthquake activity^5^,^6^ and explosive submarine eruptions^10^.

Several authors postulated a topography of the permeability boundary layer, marking the lithosphere-asthenosphere-boundary that guides melt from amagmatic segments towards magmatic centres^4^,^9^,^11^, enabling enhanced magma flux. Recent observations of maximum earthquake depths along the axes of ultraslow ridges indicate systematic variations in the lithosphere’s thermal structure^12^ that, when extrapolated to depth, outline the lithosphere-asthenosphere-boundary topography and support the concept of melt focussing beneath magmatic centres.

The easternmost portion of the Southwest Indian Ridge (SWIR; Fig. 1a) between Melville Fracture Zone and Rodriguez Triple Junction shows an anomalously low average crustal thickness of ~3 km and an unusually deep (~4.7 km) axial rift valley^9^. The thin crust and the deep axial valley imply a vastly reduced magmatism for this portion of the SWIR, which was confirmed in various studies using wide angle seismics^8^, gravity modeling^9^ and side scan sonar imagery^13^. The presence of three prominent axial highs (Segments 8, 11, 14 after ref. 9) is in contrast to the overall reduced magmatism at this SWIR portion. The axial highs show a locally thickened crust^9^ and are interpreted as isolated volcanic centres. These volcanic centres exhibit a much higher relief to length ratio and are spaced at greater distances along the ridge axis in comparison to volcanic centres at the slow spreading Mid-Atlantic Ridge^4^. These high relief volcanic centres seem to be connected with magmatic segments of lower relief (Segment 7, Fig. 1) that are proposed to have no melt regions of their own but to be laterally fed by the main volcanic centres^14^.

Numerous magmatic/volcanic centres at ultraslow-spreading ridges show off-axis bathymetric highs, oriented perpendicular to the spreading axis, that document enhanced magmatism and crustal thickness over sustained periods of time^15^. However, at the easternmost SWIR magmatism changes in space and time, as shown by the considerable variability in off-axis crustal thickness and rock type^4^,^9^. In the period from July 1996 to November 2001 several teleseismic earthquake swarms occurred at the Segment 8 (ref. 6; Fig. 1b,c) that had magnitudes of 4.3–5.5 m_b_ which is remarkably strong for mid-ocean ridge earthquakes. As the closest recording stations are far away, large location uncertainties (~20 km) prevent a detailed geological interpretation of the teleseismic events.

Studies of local earthquake activity have greatly advanced our understanding of spreading episodes at faster mid-ocean ridges^16^,^17^,^18^. Owing to the poor accessibility of ultraslow-spreading ridges comprehensive records of local seismicity did not exist until very recently^12^. In particular the processes of melt formation and migration as well as the factors that control the dynamics of volcanic centres at ultraslow ridges remain unclear, leading to the following open questions:

- At what depth does melting take place and how are melts distributed within the lithosphere?
- How does the lithosphere’s rheology and thermal structure vary at the transition from volcanic centres to amagmatic portions of the ridge?
- What is the cause of unexpectedly strong earthquakes?

Here we present the results of a local passive seismic experiment that studied a major volcanic centre and its neighbouring segment during a phase of magmatic activity.

## Results

**Figure 1:**
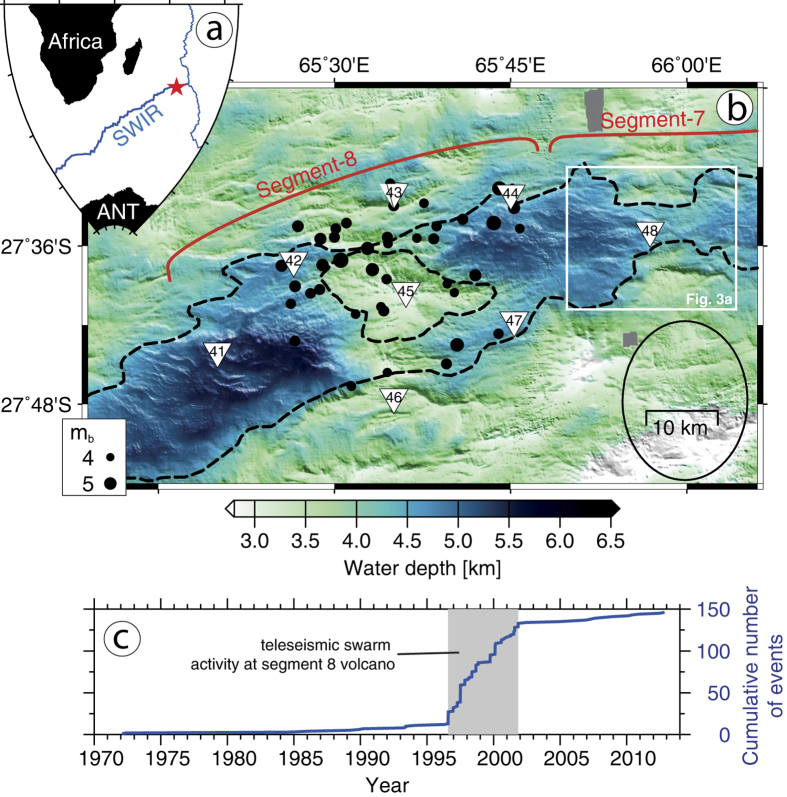
Teleseismic events between 1970–2013 at Segments 8 and 7 of the Southwest Indian Ridge (SWIR). (**a**) Location of the survey area (red star). (**b**) Bathymetry with dashed lines outlining the extent of the axial rift valley and the Segment 8 volcano. Black dots are epicentres of the EHB bulletin (http://www.isc.ac.uk/ehbbulletin) during the teleseismic swarm activity from 1996 to 2001. (**c**) Size scales with magnitude and black ellipse indicates the average location error. White square refers to map in Fig. 3a and triangles are stations deployed for the microearthquake study in 2012–2013. (**c**) Cumulative teleseismic earthquake numbers of the more comprehensive international seismological centre ISC bulletin (http://www.isc.ac.uk/iscbulletin) for the map b area. All maps and graphs were created with the GMT software^38^.

We deployed a network of eight ocean bottom seismometers around the Segment 8 volcano (Fig. 1b) that recorded microseismicity from October 2012 to August 2013. Hypocentres of 2974 local events were initially located on the basis of a 1D velocity model^12^ and give insight into the seismic activity in space and time of this SWIR segment. A total of 25,725 P- and S-wave ray paths sampling the lithosphere were used for a local earthquake tomography^19^ that images the 3D velocity structure of the volcanic centre (see Methods section).

**Figure 2:**
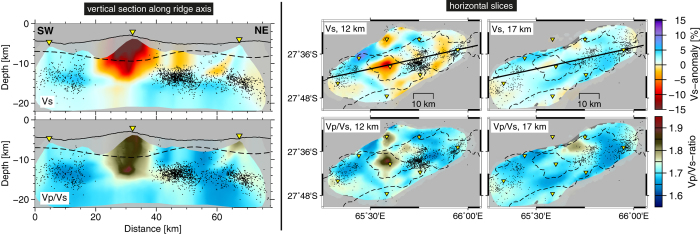
Results of local earthquake tomography. Colours indicate anomalies in the S-wave velocity Vs (upper panels) and in Vp/Vs ratio (lower panels) structure. Vertical section location is given by black solid line in horizontal slices. Black dots are projected microearthquakes as relocated during the tomography and yellow inverted triangles are projected OBS locations. Dashed line in left hand panels shows gravity derived crustal thickness from ref. 9. Dashed lines in right hand panels outline extent of the axial rift valley and the volcano, c.f. Fig. 1b. Note that areas of poor or no ray coverage that moreover have no grid nodes in the tomography model (see Supplementary Fig. 2) appear shaded in grey. All figures were created with the GMT software^38^.

A prominent aseismic zone was observed beneath the Segment 8 volcano during our microseismicity study (2012–2013) that extends about 20 km along the ridge axis (Fig. 2). In its centre the local earthquake tomography revealed a distinct anomaly of low P- and S-wave velocities (Vs) and high Vp/Vs ratio (Fig. 2). In particular the lower limit of this low velocity anomaly (LVA) at 15 km depth could be well constrained by our tomography model while lateral extent and absolute amplitude are less well recovered (see methods section and Supplementary Figs 4 and 5). East and west of the Segment 8 volcano maximum depths of microearthquakes rapidly increase to ~15 km.

**Figure 3:**
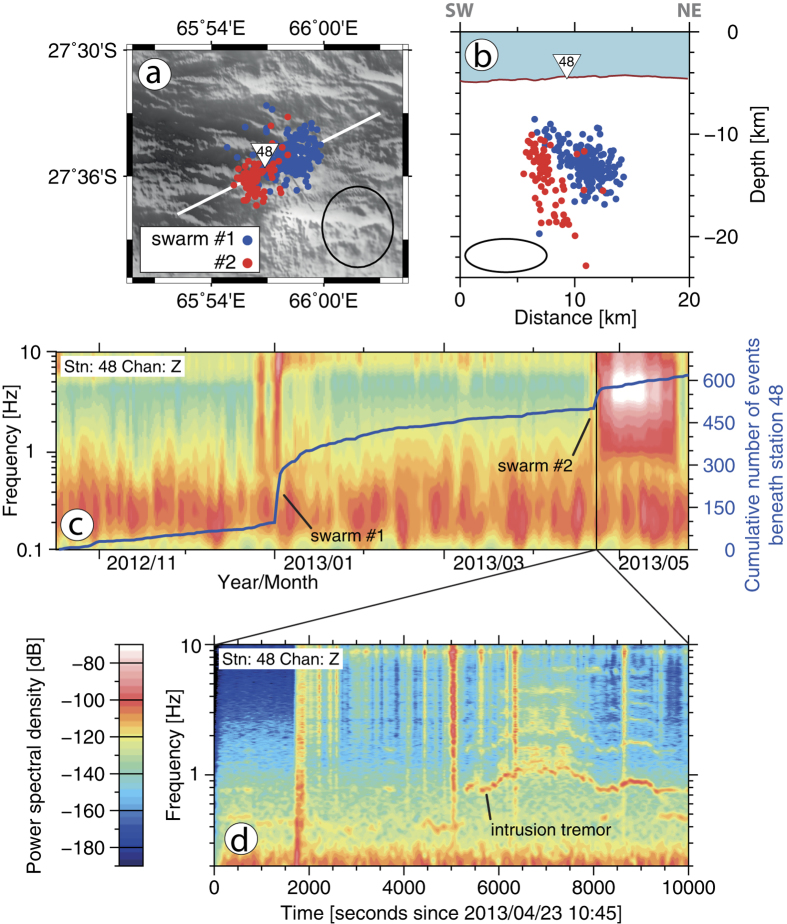
(**a,b**) Map view and cross-section showing the location of microearthquake swarms below SWIR Segment 7 (ref. 9). Black ellipses represent the average location error. (**c**) Cumulative number of events beneath station 48 (blue curve) and power spectral density for the entire survey period. (**d**) Close-up spectrogram showing swarm #2 events (vertical lines) and the onset of harmonic intrusion tremor. All figures were created with the GMT software^38^.

Two distinct swarms of microearthquakes occurred in January and April 2013 below the centre of SWIR Segment 7 (ref. 9; Fig. 3a,b) which is located ~35 km east of the Segment 8 volcano. Here the axial rift valley becomes shallower and narrower (Fig. 1b) and the crustal thickness increases again (Fig. 2). These microearthquake swarms lasted for few days each and located at depths of 8–20 km beneath station 48 that ensures a good depth control for the swarm hypocentres (Fig. 3a,b). The swarms occurred in close spatial proximity to each other. Soon after the first event of swarm #2 we observe the onset of an intrusion tremor (Fig. 3c,d) that is exclusively recorded at station 48. The tremor contains most of its energy in a frequency band around 1 Hz that exhibits frequency gliding and is accompanied by several harmonics. Tremors of similar characteristics are commonly recorded at active volcanoes^20^ and prior to eruptions^21^.

Owing to their spatial and temporal proximity the microearthquake swarms and the intrusion tremor strongly suggest a dyking episode associated with magma movement beneath Segment 7 in 2013.

## Discussion

It is a common issue in passive source tomography that amplitudes of model anomalies depend on the ray coverage and damping parameters. Synthetic tests with checkerboard patters or custom shaped realistic anomalies represent the best way to estimate the effects of smearing and damping, and to assess their representation of true amplitudes. Therefore, the conversion of seismic velocities and their derivatives into physical properties such as temperature and melt content may be ambiguous and unwarranted. Accordingly, we refrain from calculating temperature and melt content but focus our interpretation on the relative amplitude and shape of the anomalies.

The region inside the aseismic zone below the volcano is of particular interest to our study. Here, in the 10–20 km depth range there is ample ray coverage (Supplementary Fig. 2) but at shallower depth the geometry and density of rays is less favourable. A synthetic test, comprising a realistic low velocity anomaly in the centre of the aseismic zone (test #3 in methods section, Supplementary Fig. 5) showed that in this region of the model anomalies may be smeared out horizontally at the lower end and amplitudes are likely underestimated. The LVAs lower end that lies in a region of good ray coverage is well beneath the gravity derived lower boundary of the crust (Fig. 2).

Considering the potential underestimation of velocity anomalies inside the aseismic zone and Vp/Vs ratios reaching 1.9 in the centre of the LVA, these values may represent the lower limit of the true Vp/Vs ratio in the lithosphere. Melts in upper mantle rocks are typically associated with Vp/Vs ratios of 1.8–2.1 depending on the fraction of melt^22^. We therefore conclude that the observed Vp/Vs ratio inside the LVA requires at least partially the presence of melts and hence presents evidence for a melt body beneath the Segment 8 volcano that extends down to 15 km depth. For the upper part of the LVA other fluids or water filling cracks and pore spaces might additionally contribute to the velocity anomaly^23^.

### Reconstruction of a magmatic spreading episode

Based on our findings we are not only able to illuminate the current structure of the axial lithosphere below the Segment 8 volcano but, additionally, these rare *in-situ* observations enable a reconstruction of essential stages of a magmatic spreading episode at an ultraslow-spreading ridge.

The current spreading episode was preceded by a phase of intense seismic activity. At least 7 teleseismic earthquake swarms centred on the Segment 8 (Fig. 1b) occurred in the period 1996–2001 (Fig. 1c) with magnitudes of 4.3–5.5 m_b_, exceeding by far the usual background seismicity along the SWIR^6^. Focal mechanisms of the teleseismic swarm events indicate the failure of rift parallel normal faults^24^. We infer that these earthquake swarms marked the beginning of an extended phase of magmatic activity that continued throughout our survey period, hence lasting over a decade. At the Gakkel Ridge (Arctic Ocean), a large teleseismic earthquake swarm also occurred at the onset of a magmatic phase of at least 2 years duration^10^,^25^. The strong seismicity that appears to be associated with spreading episodes at the massive volcanic centres of ultraslow spreading ridges may either be triggered by the ascent of mantle melts or it may in turn facilitate their way through the lithosphere.

**Figure 4:**
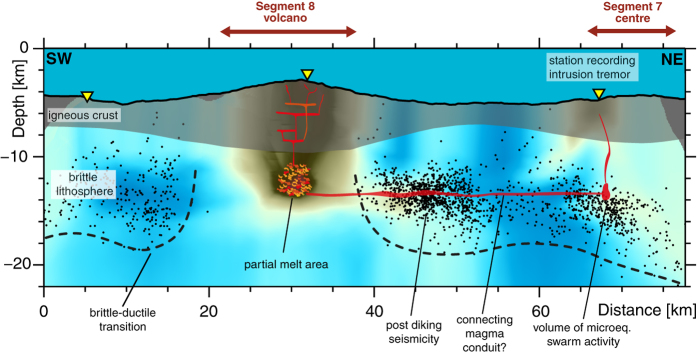
Conceptual sketch illustrating the lithospheric structure and the magma plumbing system below the Segment 8 volcano. Background displays Vp/Vs ratio of the tomography model (c.f. Fig. 2) and black dots are projected microearthquakes. Red polygons indicate locations of conceivable magma bearing sills and dykes. LVA: low velocity anomaly. Note that sketched sills and dikes are purely speculative as they might have a smaller extent than the tomography models resolution capability and are therefore beyond imaging resolution. All figures were created with the GMT software^38^.

In 2012/2013, an area devoid of earthquakes extends 20 km along the ridge axis below the Segment 8 volcano, suggesting that in this area temperatures exceed those of brittle deformation. Since most of the teleseismic events in 1996–2001 locate in the same area (Fig. 1a), the mechanical strength and hence thermal structure must have considerably changed between 2001 and the start of our microearthquake survey in 2012 suggesting the area was heated up meanwhile. Inside this aseismic zone the tomography model shows a prominent LVA that we interpret as a reservoir containing partial melts (Figs 2 and 4). We propose that between 1996 and 2012 this reservoir of partial melts has either re-grown or was newly formed beneath the Segment 8 volcano that represents the backbone of the magma plumbing system present in 2012/2013 (Fig. 4). Its depth extends well into the mantle and is much deeper than the axial melt lenses that have been imaged by active or passive seismics at faster spreading ridges.

The tomography model does not show a low velocity, high Vp/Vs ratio anomaly indicative for the presence of melts below the volume of microearthquake swarm activity (Fig. 4) in the centre of Segment 7 although ray coverage is sufficient here (Supplementary Fig. 2). We conclude that the LVA below the Segment 8 volcano is the only stable melt reservoir at that time in the study area.

As we observed dyking at Segment 7 during our experiment we hypothesize that the melt reservoir beneath Segment 8 may laterally feed magma to Segment 7. The lateral movement of melt in the lithosphere over distances larger 30 km is none unique to our study and has been observed in various rift zones e.g. the Afar rift^26^ or the Bárðarbunga volcanic system^27^. It has further been postulated to explain the difference in morphology between high-relief volcanic segments and their accompanying low-relief neighbouring segments^14^.

However, our tomographic model cannot resolve where and at what depth-level lateral magma feeding occurs. The width of a potential connecting magma conduit may be in the order of a few meters to tens of meters, according to geodetic observations at comparable terrestrial rifts on Iceland and the Afar spreading centre^26^,^27^,^28^. This is below the minimum feature size our tomography model can image (see Supplement Figs 3 and 4), making it “invisible”.

A potential indicator for the cross-feeding of magma between Segments 8 and 7 may be the cluster of enhanced microseismicity occurring immediately east of the aseismic zone (at km 40–55 in Fig. 4) that may be associated with post-dyking stress release in the lithosphere, a phenomenon that was previously observed in actively dyking rift systems^16^,^29^.

Our observations show that the spreading episode at SWIR Segment 8 lasted for at least 12 years, and it may still be ongoing. The Segment 8 volcanic centre finds an analogue in the 85°E volcanic complex at the ultraslow-spreading Gakkel Ridge (Arctic Ocean) that yielded a teleseismic earthquake swarm of unprecedented length and magnitude in 1999 (ref. 5). This swarm was partly associated with a series of small volume eruptions of both effusive and explosive character, as indicated by the recording of explosion sounds in 2001 (ref. 25) and the discovery of fresh lava flows and pyroclastic deposits covering the sea floor, around the volcanic complex^10^.

A 16 days long study of local earthquake activity in 2007, based on seismometers deployed on the sea ice, found hypocentres down to 13 km beneath the sea floor at the sites axial volcanoes^30^. This showed that the lithosphere under the 85° E volcanic complex had cooled and the spreading episode had potentially terminated, after lasting 8 year at maximum.

## Conclusions

The estimated ~10,000 yr (ref. 31) recurrence cycle of eruptions at volcanic centres on ultraslow-spreading rates is possibly the lowest for all mid-ocean ridge types, diminishing the chance to directly observe these eruptions. This makes the records from the 85°E volcanic complex at the Gakkel Ridge^5^,^10^,^30^ and the more detailed observations from the SWIR Segment 8 volcano presented herein especially invaluable to understand the nature of such volcanic systems. Our study presents the first image of a melt reservoir at mantle depths below the axis of an ultraslow spreading ridge during a phase of magmatic activity. At faster spreading ridges where shallow lithospheric melt regions are more common, eruptions occur frequently. There, much more detailed reconstructions of volcanic episodes have been possible^32^ but they refer to spreading processes that differ greatly from the magma poor conditions at ultraslow spreading ridges.

Presuming the initiation of the spreading episode at the Segment 8 was associated with enhanced earthquake activity in 1996–2001 it lasted well over a decade. This would be the longest so far recorded spreading episode at any mid-ocean ridge^16^,^17^. Spreading episodes at faster ridges are shorter and may only be detected locally as their seismic activity is of weak magnitudes.

The transition in the seismic activity at Segment 8, from hosting m_b_ 5.5 earthquakes in 1996–2001 to an aseismic zone in 2012/2013, indicates that the thermal structure of the lithosphere below volcanic centres at ultraslow-spreading ridges may considerably alter between phases of quiescence and spreading episodes. The brittle lithosphere may be cold and thick during phases of quiescence but the ascent of large quantities of melt at the onset of spreading episodes causes the lithosphere to heat up and alter its rheology from brittle to ductile, as observed under the Segment 8 volcano. Once a magma plumbing system is established it may host large enough amounts of melt to possibly feed neighbouring ridge segments through lateral conduits as we postulate for SWIR Segments 8 and 7.

We conclude that spreading episodes at ultraslow mid-ocean ridges are rare but may last over years to decades – typically initiated by strong tectonic earthquakes^6^. They include the establishment of a deep reaching reservoir under the high-relief volcanic centres that may supply melts to neighbouring, less prominent volcanic segments.

## Methods

### Microearthquake data processing

Seismic data were recorded by eight free-fall ocean bottom seismometers equipped with Güralp CMG-40T broadband sensors and HiTech Inc hydrophones deployed on October 17, 2012 (Fig. 1b). Seismic records of individual stations span 7–10 month depending on battery capacity. The recorder clock drift was corrected, assuming a linear drift during the recording interval, with the method of ref. 33 and taking station 47 as reference station. Event identification in the waveforms, phase onset picking and the hypocentre location based on a 1D velocity model are documented in ref. 12. Pick uncertainties were estimated during the manual picking procedure and have averages of ±0.07 s and ±0.11 s for P- and S-phases, respectively. S-phases were generally picked on horizontal channels, except for stations 42, and 45 where horizontal channels were malfunctioning.

### Spectrogram analysis

Prior to signal processing the instrument response was removed from the waveforms. Time series of daily averages of power spectral density for the entire seismic record of station 48 (Fig. 3c) were calculated with the PDFSA software package of ref. 34. The close-up spectrogram of the intrusion tremor (Fig. 3d) was calculated with the ObsPy software^35^. For the calculation of spectrograms, data were bandpass filtered at 0.01–50 Hz. Amplitude spectra were calculated for 60 s windows that overlap by 10%.

### Local earthquake tomography

Events for local earthquake tomographic inversion were selected from the earthquake catalogue of ref. 12. Sources far outside the network (i.e. events further than 20 km i.e. one hypocentre depth beyond the network) produce rays that sample the lithosphere outside the network and might drag anomalies from beyond to within of the network. To retain a maximum number of rays, while excluding poorly located events from outside of the network, we applied the following event selection criteria: Events inside the network must contain at least 7 phase onset picks. Events outside the network must not be further away than 20 km from the nearest station and have at least 10 phase picks. All other events were omitted.

In total we obtained 25,725 arrival times (12,491*P*; 13,234*S*) as input data that originate from 2365 events. We used the LOTOS iterative least squares tomography algorithm^19^ which can simultaneously invert for the *P*- and *S*-wave velocity structures of the lithosphere and the source parameters of earthquakes. The tomography is commenced with an initial source location based on a 1D velocity model and straight ray paths. We used the starting 1D *P*-wave velocity model of ref. 12 but included slightly lower velocities in the depth interval at 7–15 km below sea level (Supplementary Fig. 1a). The best fitting 1D model of ref. 12 applies for an extended along axis region. When used as initial model for the tomographic inversion of the area inside the network that covers mainly the volcanic complex it produces an extended low velocity anomaly in centre of the model output suggesting that the background velocity model as such is slightly too fast.

We started with slower velocities (Supplementary Fig. 1) that better represent the velocity structure beneath the volcanic complex. *S*-wave velocities of the 1D starting model were calculated from the Vp model assuming a constant Vp/Vs ratio of 1.73, which corresponds to a poisson ratio of 0.25, common for igneous rocks.

For the tomography the following steps were performed:

- Location of sources in the 3D velocity model utilizing the bending tracing algorithm of ref. 36. In this step the sea floor topography is implemented so that rays and sources in the water column are not allowed.
- Construction of model grids with nodes spaced at 2 km in horizontal directions and a variable vertical spacing (Supplementary Fig. 2). The vertical node spacing is 1 km in areas of dense ray coverage and is sparse in areas of less ray coverage. Velocities are linearly interpolated between grid nodes.
- To overcome any grid related artefacts in the tomography models the inversion was performed for several grids at different azimuthal orientations of 0°, 22°, 45°, 67° and results were averaged afterwards.
- The actual matrix inversion was performed in a least squares manner using the LSQR algorithm of ref. 37. To achieve a stable solution we applied the fattening damping by minimizing the velocity anomaly difference between neighbouring nodes. Weights for damping and source correction were determined based on the results of synthetic tests.

The steps of source location, matrix calculation and inversion were successively repeated three times for all tomography models (experimental data- and synthetic data cases). Residuals did not substantially decrease after the third iteration. The parameterisation grids were constructed in the first iteration; then the velocity values were updated at the same grid nodes. Damping and weighting parameters for the tomographic inversion were selected from the optimum parameters of synthetic recovery tests. A station correction was not necessary since we did not observe systematic residuals that call for such a correction.

### Synthetic testing

We created a series of synthetic tests to benchmark the results of the local earthquake tomography and to estimate optimum weighting and damping parameters. The station- and earthquake source locations were identical to the last iteration of the real data case. Synthetic travel times were computed via 3D ray tracing and afterwards all structural information and source coordinates were “forgotten”. Additionally, the travel time residuals resulting from the third iteration of the real data case were multiplied with a factor of 0.2 and the product was added to the synthetic travel times to incorporate the effect of noise. The tomographic inversion was then performed in the same manner as for the real data case.

Test #1 represents a checkerboard of vertical prisms at various sizes with ±7% alternating velocity anomalies having opposite signs for P- and S-wave models (Supplementary Fig. 3). The main purpose of the test is to explore the horizontal resolution capability of the tomographic model. Synthetic anomalies are generally better recovered at 12 km than at 17 km depth. Larger anomalies are better restored than smaller ones both in structure and amplitude. In particular the anomalies of 3 × 3 km size appear blurred, delineating the lower resolution limit of our model.

Structure and amplitude are better resolved in areas where sources are present and anomalies become smeared in areas devoid of sources. The smearing of anomalies reflects the intrinsic trade-off between the velocity- and source parameters. The specific geometry of sources and receivers has an impact on the smearing and the amplitude of restored anomalies. Thus, the results of the synthetic tests provide a realistic representation of the tomography model’s capability to recover the true velocity structure.

Test #2 comprises horizontal rectangular prisms at different sizes that are oriented perpendicular to a vertical cross-section along the ridge axis and have alternating anomalies of ±7% (Supplementary Fig. 4). This test explores the vertical resolution capability of the tomography model. The general structure of the input model could be recovered within the network. As in test #1 we observed a slightly better resolution for areas where sources are present (Supplementary Fig. 4). Anomalies in the upper row are smeared towards the sea floor since the majority of rays at these depths are near vertical (Supplementary Fig. 2) and sources are scarce. Within the aseismic zone beneath the volcano, the general structure could be resolved although amplitudes remain weaker than in the input model.

Test #3 represents a realistic low velocity/high Vp/Vs ratio body in the centre of the aseismic zone (Supplementary Fig. 5) in analogy to the LVA in the real data model (Fig. 2.). The sparse ray coverage in some parts of the aseismic zone (Supplementary Fig. 2) questions the trustworthiness of the LVA in the final velocity model (Fig. 2). The test intends to verify the tomography model inside the aseismic zone. The vertical extent of the anomaly was well recovered, in particular at the lower boundary. As rays bundle beneath the station on top of the volcano, the horizontal dimension of the anomaly could not be fully restored in this area. At its lower end the anomaly appears horizontally smeared due to the near horizontal alignment of rays here. The amplitude of the recovered anomaly remains lower than in the input model due to smoothing during the tomographic inversion. The results of the synthetic test suggest that the LVA inside the aseismic zone likely has a smaller horizontal extent at its base (due to smearing) and its amplitude is possibly underestimated, whereas the depth extent to 15 km is well resolved.

Large positive travel time residuals observed at the station on top of the volcano (station 45), after location with a 1D velocity model^12^, compared to travel time residuals at other stations further support the existence of a LVA inside the aseismic zone (see Supplementary Fig. 6a). There is no sedimentary cover on the volcano^13^ that could produce such a delay. The delay must therefore originate from a low velocity anomaly in the crust or upper mantle. To asses the effect of the synthetic anomaly in test #3 we compared the travel times for this synthetic model with those of an anomaly free synthetic model (Supplementary Fig. 6c). The comparison clearly shows that the anomaly accounts for about 150 ms of the S-phase delay at station 45 while it does not affect the remaining stations. Omitting this anomaly in the final model would leave a residual of 150 ms at station 45, which also exceeds the picking uncertainty of S phases (±110 ms). We conclude that the delayed S-phases at station 45 require the presence of a LVA inside the aseismic zone.

## Additional Information

**How to cite this article**: Schmid, F. *et al*. Magma plumbing system and seismicity of an active mid-ocean ridge volcano. *Sci. Rep.* **7**, 42949; doi: 10.1038/srep42949 (2017).

**Publisher's note:** Springer Nature remains neutral with regard to jurisdictional claims in published maps and institutional affiliations.

## Supplementary Material

Supplementary Information
