# Supplementary material for: Magma plumbing system and seismicity of an active mid-ocean ridge volcano
Source: Sci Rep. 2017 Feb 20;7:42949. doi: 10.1038/srep42949 (PMC5317165; doi:10.1038/srep42949)
Supplement: Supplementary Information [file srep42949-s1.pdf]

Supplementary information to the manuscript

Magma plumbing system and seismicity of an active mid-ocean ridge volcano

By Florian Schmid*, Vera Schlindwein, Ivan Koulakov, Aline Plötz, John-Robert Scholz

*Corresponding author, fschmid@awi.de

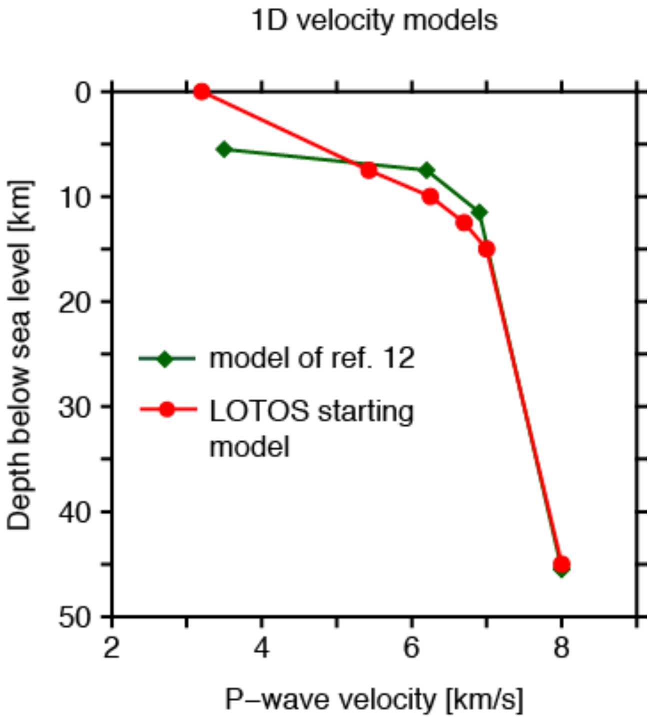

Supplementary Figure 1. *Starting 1D P-wave velocity model for the local earthquake tomography (red). Note that the 1D model of ref.* [13] *(green) is only defined below the deepest OBS station (at 4.461 km). Symbols mark defining velocity nodes in each model.*

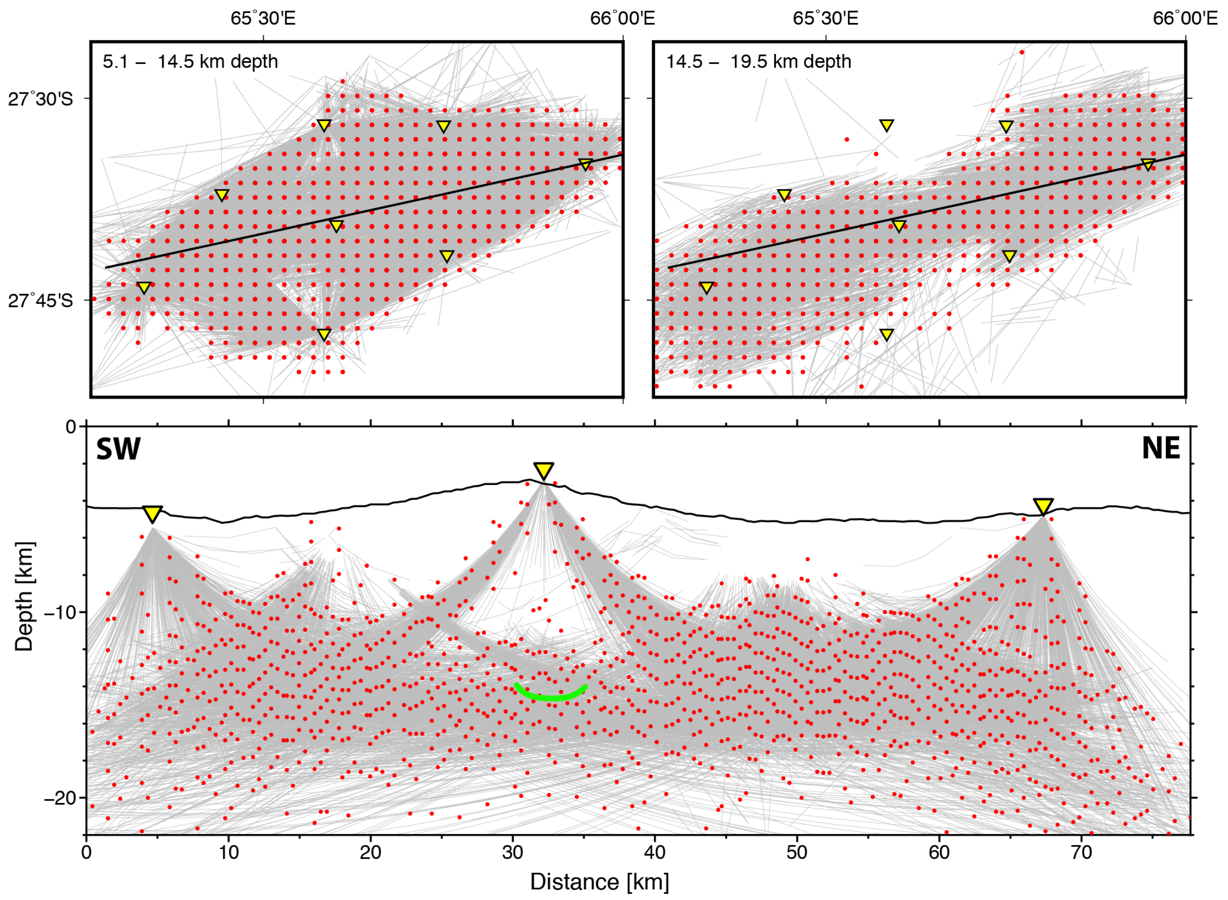

Supplementary Figure 2. *Ray paths (gray lines) and grid nodes (red points) of the tomography model. Upper panels are maps with projected rays and nodes for the given depth interval. Solid black lines indicates the position of the lower panel cross-section. All rays and nodes closer than 5 km to the cross-section are projected. The map and cross-section locations are identical Fig. 2 in the manuscript. The green line indicates the lower boundary of the low velocity anomaly beneath the volcano. Note that the vertical distribution of grid nodes varies depending on the density of rays.*

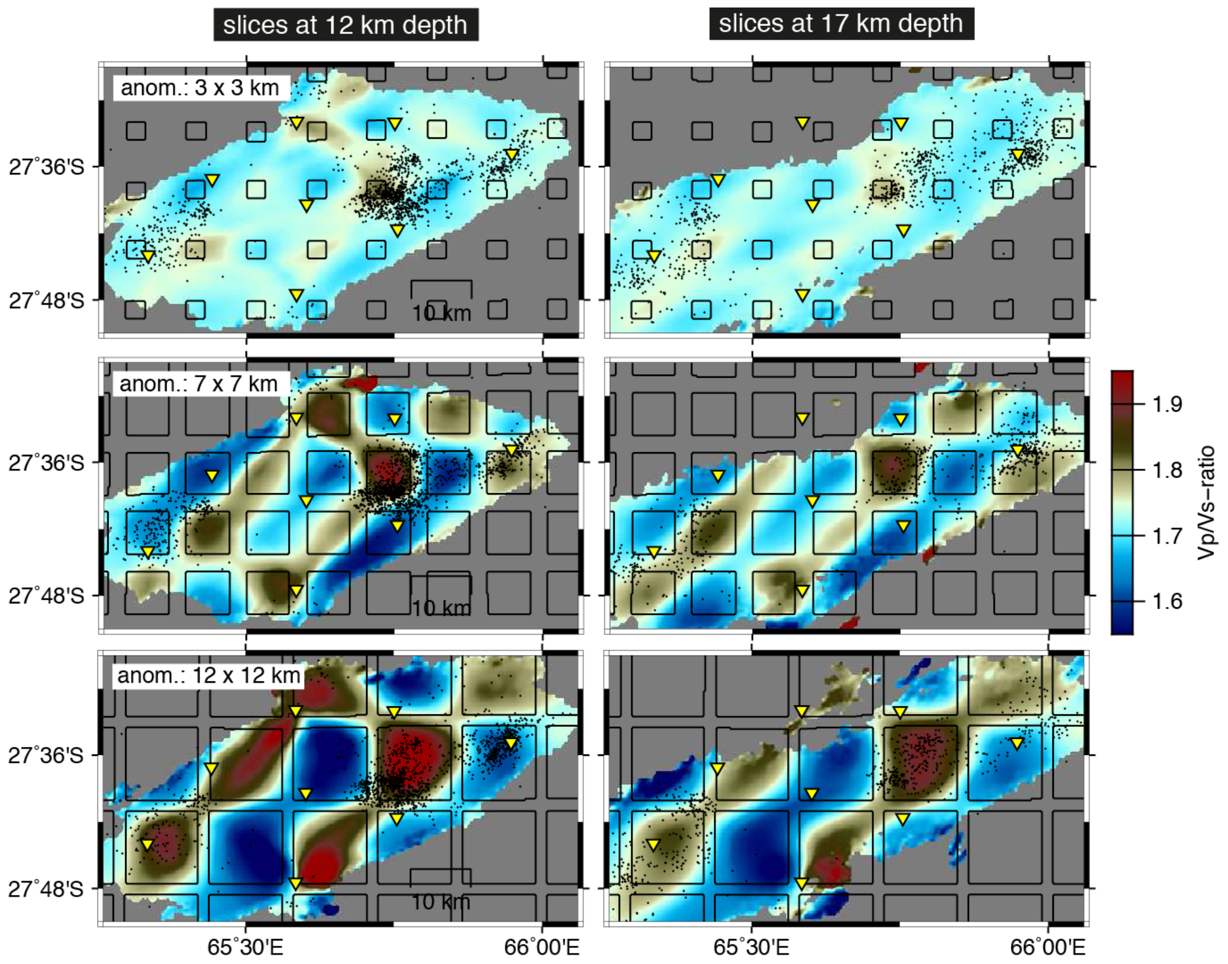

Supplementary Figure 3. *Synthetic test #1 horizontal slices of the input model and recovered anomalies. Inverted triangles are receivers. Black dots show source locations. Black squares represent the input model pattern. Note, that the velocity perturbations of the input model are equal for the three models of different prism size.*

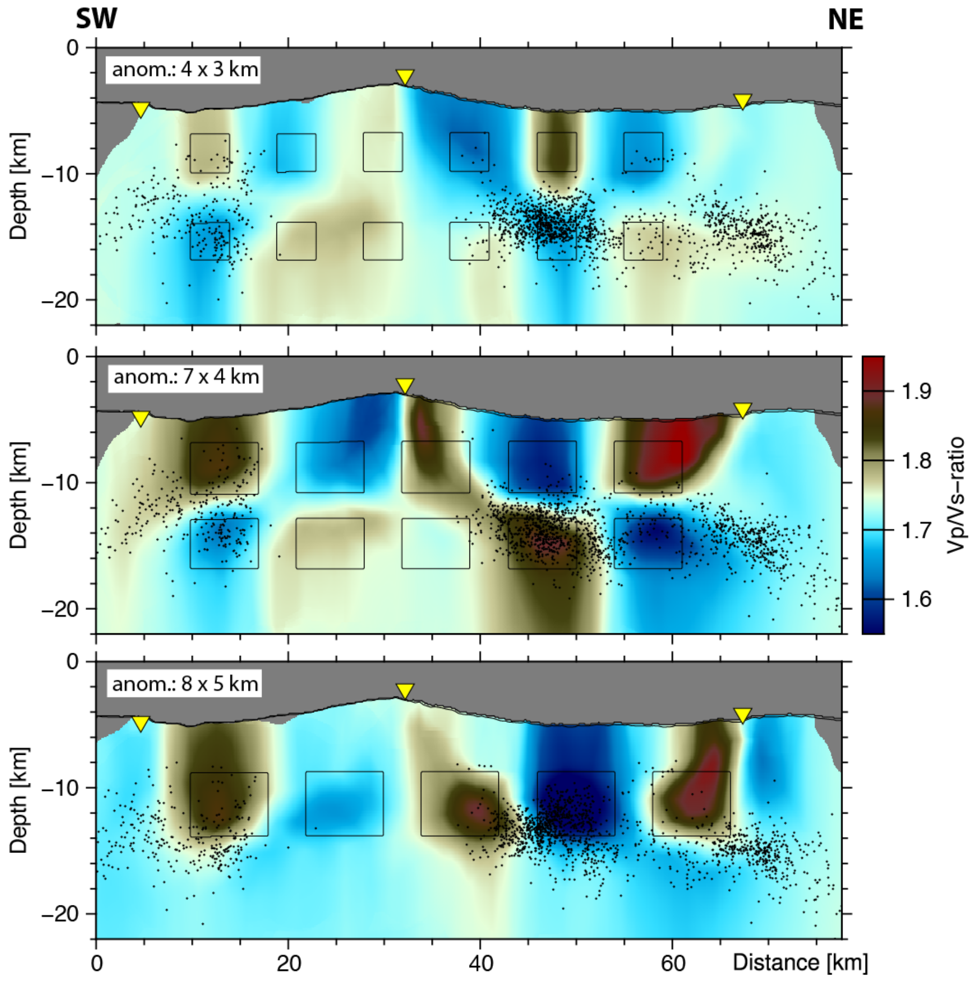

Supplementary Figure 4. *Vertical sections of synthetic test #2 input model and recovered anomalies. The location of the displayed cross-section is equal to that of Figs. 2 and 3 in the manuscript. Black squares represent the horizontal prisms of the input model. Note, that the velocity perturbations of the input model are equal for the three models of different prism size.*

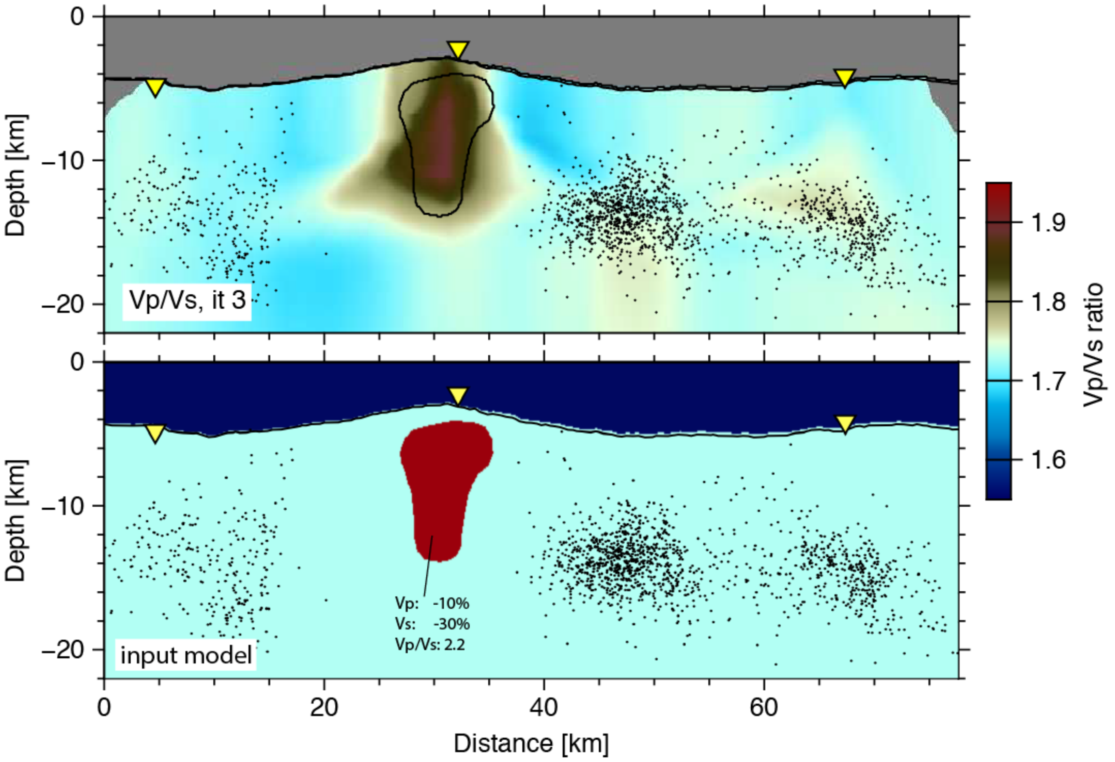

Supplementary Figure 5. *Synthetic test #3 with a realistic low velocity body in the centre of the aseismic zone. The displayed cross-section is identical to the previous figures. The upper panel shows the recovered Vp/Vs ratio. The lower panel shows the input model.*

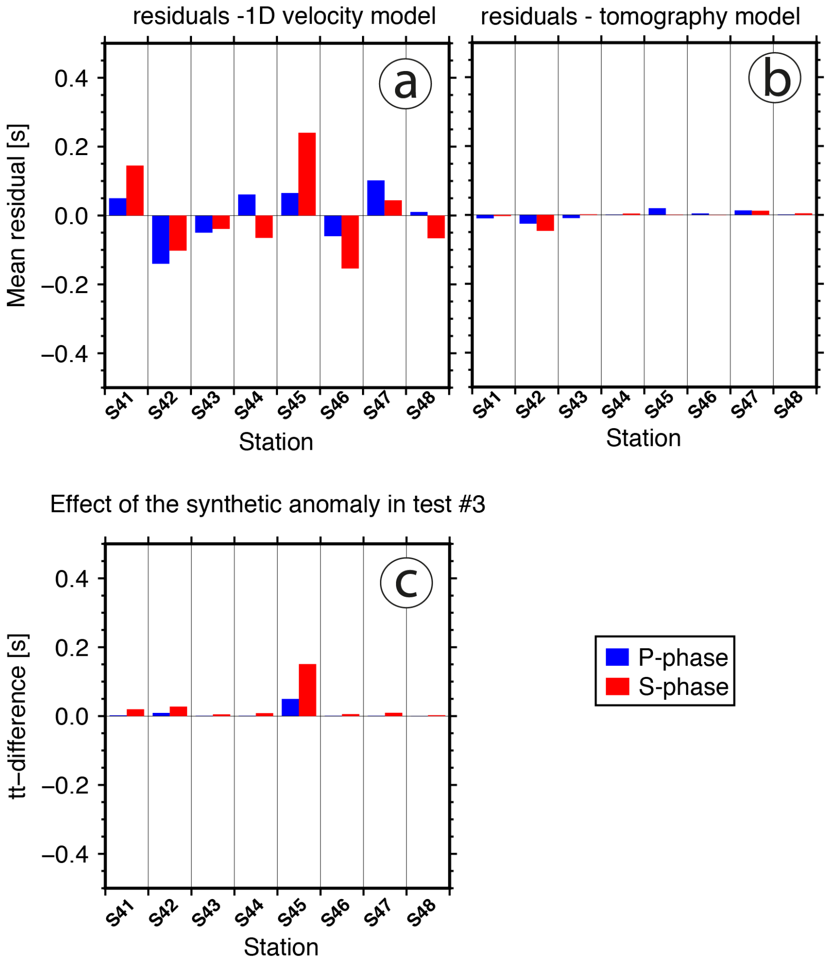

***Supplementary Figure 6. a**, Travel time residuals at individual stations after locating the events selected for the local earthquake tomography with a 1D velocity model in the HYPOSAT software*[37] *(see also ref. 12). **b**, Travel time residuals for individual stations of the final tomography model. **c**, Differences in travel times computed by ray tracing through synthetic model without anomalies (uniform 1D velocity distribution) and synthetic model #3. Note that random noise was added to the travel times.*

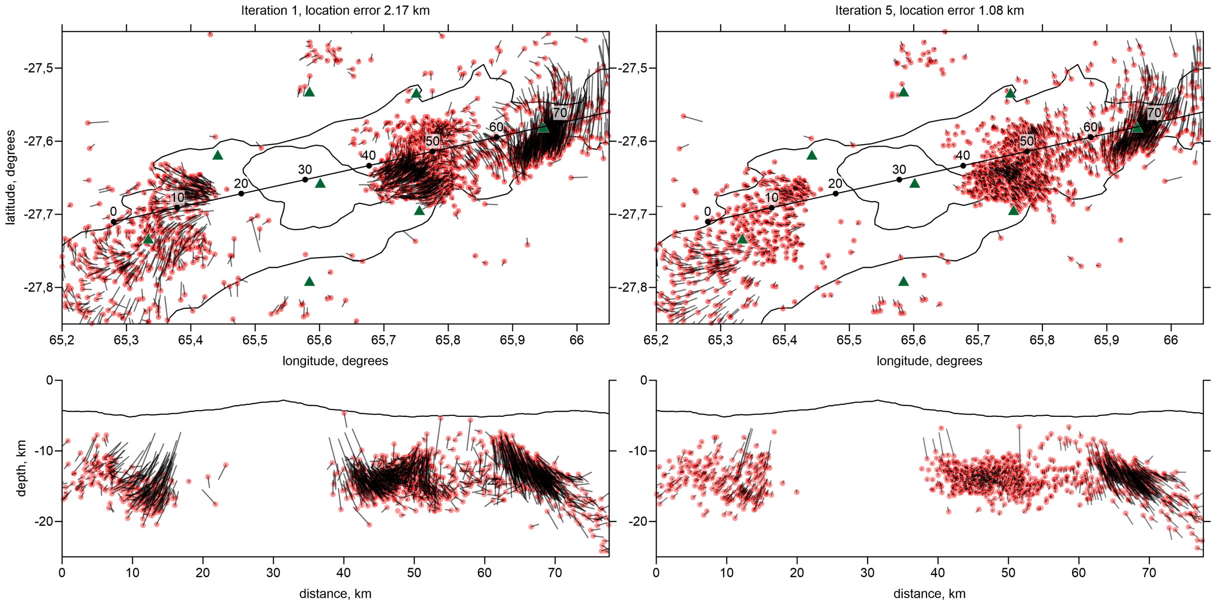

Supplementary Figure *7. Source mislocations in the synthetic test #2 with checkerboard anomalies of 12x12 km extent (see supplementary Fig. 4). Left panels present the results of source locations in the starting one-dimensional velocity model, whereas the right plots present the locations results in the final 3D model. Upper panels present the source locations in the map view and the lower panels show projections sources (location of the profile is shown in the map). Red dots depict the current locations, and the bars direct to the true source points. Green triangles indicate the seismic stations.*

Additional References

37 Schweitzer, J. (2001), HYPOSAT—An enhanced routine to locate seismic events, Pure Appl. Geophys., 158, 277–89, doi:10.1007/PL00001160.
